# Unhappiness and dissatisfaction in doctors cannot be predicted by selectors from medical school application forms: A prospective, longitudinal study

**DOI:** 10.1186/1472-6920-5-38

**Published:** 2005-12-13

**Authors:** IC McManus, Sheeraz Iqbal, Amuthan Chandrarajan, E Ferguson, Joanna Leaviss

**Affiliations:** 1 Department of Psychology, University College London, Gower Street, London WC1E 6BT, UK; 2 Department of Psychology, University College London, Gower Street, London WC1E 6BT, UK; 3 Department of Psychology, University College London, Gower Street, London WC1E 6BT, UK; 4 School of Psychology, University of Nottingham, Nottingham NG7 2RD, UK; 5 School of Psychology, University of Nottingham, Nottingham NG7 2RD, UK

## Abstract

**Background:**

Personal statements and referees' reports are widely used on medical school application forms, particularly in the UK, to assess the suitability of candidates for a career in medicine. However there are few studies which assess the validity of such information for predicting unhappiness or dissatisfaction with a career in medicine. Here we combine data from a long-term prospective study of medical student selection and training, with an experimental approach in which a large number of assessors used a paired comparison technique to predict outcome.

**Methods:**

Data from a large-scale prospective study of students applying to UK medical schools in 1990 were used to identify 40 pairs of doctors, matched by sex, for whom personal statements and referees' reports were available, and who in a 2002/3 follow-up study, one pair member was very satisfied and the other very dissatisfied with medicine as a career. In 2005, 96 assessors, who were experienced medical school selectors, doctors, medical students or psychology students, used information from the doctors' original applications to judge which member of each pair of doctors was the happier, more satisfied doctor.

**Results:**

None of the groups of assessors were significantly different from chance expectations in using applicants' personal statements and the referees' reports to predict actual future satisfaction or dissatisfaction, the distribution being similar to binomial expectations. However judgements of pairs of application forms from pairs of doctors showed a non-binomial distribution, indicating consensus among assessors as to which doctor would be the happy doctor (although the consensus was wrong in half the cases). Assessors taking longer to do the task concurred more. Consensus judgements seem mainly to be based on referees' predictions of academic achievement (even though academic achievement is not actually a valid predictor of happiness or satisfaction).

**Conclusion:**

Although widely used in medical student selection to assess motivation, interest and commitment to a medical career, the personal statement and the referee's report cannot validly be used by assessors, including experienced medical school selectors, to identify doctors who will subsequently be dissatisfied with a medical career.

## Background

Many doctors in the UK, and probably elesewhere, are unhappy with their careers in medicine, with a fifth or more of junior doctors considering leaving medicine [1]. Medical school selectors do not wish only to select academically able students who can cope academically with the medical course, but also want to assess motivation for studying medicine, becoming a doctor, and for a lifetime of practising medicine. An important question, therefore, is whether the information available during student selection can be used to predict which doctors will be happy or unhappy with a career in medicine.

Applicants to UK medical schools apply by means of the UCAS (previously UCCA) form, which contains standard demographic data, information on educational achievement and qualifications, a personal statement from the applicant, and a report from a referee [2]. Empirically, educational achievement and intellectual ability have been found to be unrelated to stress, burnout and dissatisfaction with medicine [3], and therefore the most likely source for information which might be able to predict happiness with a medical career is the applicants' personal statements and their referees' reports.

Experimental research manipulating the informational content of personal statements, resumes and cover letters shows that applicants are judged as more competent and to have greater potential when the resumes contained relevant educational references [4], whereas trying to present oneself in a positive light was not always beneficial [4,5]. Although informative, such studies do not address the actual predictive utility of personal statements, and whether selectors can identify those applicants who will eventually be successful or happy in their career, particularly when UCAS/UCCA personal statements are not structured to help selectors make their judgments.

In this study we used a simple experimental design to assess the ability of assessors to distinguish pairs of doctors who had been qualified for five or six years, one of whom who was very happy and satisfied with their medical career and the other was very unhappy and dissatisfied, using only the personal statement and referee's report submitted as a part of the doctors' UCCA application forms to medical school, twelve years previously.

## Methods

This study was approved under the normal procedures of the Ethics Committee of the UCL Department of Psychology. The study was exempt from the need for specific permission by the UCL Research Ethics Committee (see ; ).

### Design

The study used a paired comparison (two-alternative, forced-choice) design in which a group of assessors in 2005 decided which of two doctors, one very happy and one very unhappy, was the happier doctor (assessed in 2002/3), based on the personal statement and referee's report from the application forms the doctors had submitted to UCCA in 1990.

### The doctors and their application forms

**Figure 1:**
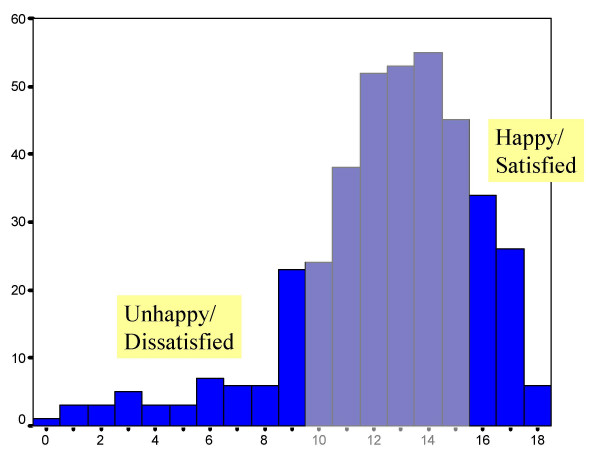
Distribution of Satisfaction scores in the doctors for whom transcribed UCCA forms were available. Doctors with a score of 16 or above were in the high satisfaction group, and those with a score of 9 or below were in the low satisfaction group.

The doctors were part of the 1991 Cohort Study and had applied to medical school during the autumn of 1990 for admission in October 1991 [6], when a wide range of measures was collected, including O-level/GCSE results, predicted A-level grades and actual A-level grades. In 2002/3 these doctors had taken part in a follow-up study which amongst other things assessed happiness and satisfaction with a medical career, as well as stress and burnout [7]; the doctors in the 2002/3 study also completed a brief Big Five personality measure [7]. The present study considered only those doctors who had returned questionnaires in the follow-up study carried out in December 2002, and for whom transcribed UCCA forms were also available. Satisfaction with medicine was assessed using a composite satisfaction score derived from three separate, temporally-anchored questions (see table 1), which correlated strongly with measures of stress and burnout (see Additional File 1). Figure 1 shows the distribution of satisfaction scores; happy doctors were classified as those scoring 16 or above, whereas unhappy doctors were those scoring 9 or below.

**Table 1:** Responses of happy and unhappy doctors.

How often do the following statements describe the way you feel about working as a doctor?	Group	Every day	A few times a week	Once a week	A few times a month	Once a month or less	A few times a year	Never
a) I think of giving up medicine for another career	**Happy**						**6**	**34**
	*Unhappy*	*17*	*8*	*5*	*9*		*1*	
b) I reflect on the satisfaction that I get from being a doctor	**Happy**	12	**25**	**3**				
	*Unhappy*		*1*		*11*	*12*	*12*	*4*
c) I regret my decision to have become a doctor	**Happy**						**5**	**35**
	*Unhappy*	*5*	*11*	*5*	*11*	*7*	*1*	

The three questions used for assessing happiness and satisfaction in the doctors. For the first and third questions (a and c) a response of 'every day' is scored as 0, 'A few times a year' as 1, through to 'Never' being scored as 6, whereas the second question (b) is scored in the reverse order. The responses of the 40 happy doctors are shown in bold, whereas the responses of the 40 unhappy doctors are shown in italics.

### Questionnaire booklets

The assessors made their judgements in questionnaire booklets which contained twenty pairs of UCCA forms, with each side of a double-page spread showing the personal statement and referee's report from a satisfied and a dissatisfied doctor, randomised to left (labelled A) or right (labelled B). Pairs were matched for sex, and were from applicants who were UK nationals aged under 21 when they applied to medical school. All forms were transcribed, checked against the originals, fully anonymised and printed in a standard format. For each pair of forms, there was a question at the bottom of the page: **"Which is the satisfied, happy doctor? ***Definitely A/Probably A/Probably B/Definitely B*". For the main statistical analysis, judgements of 'definitely' and 'probably' were combined; for correlations with the use of 'definite' judgements, see Additional File 1. Two separate booklets were assembled, Book 1 and Book 2, each containing 20 different pairs of doctors. Overall therefore, forms from 80 doctors were used, 40 being very satisfied and 40 very dissatisfied, but, in order not to overload assessors, each individual assessor saw only one booklet containing 20 pairs of forms from a total of 40 doctors. It should be emphasised that we made no attempt to differentiate 'happiness' and 'satisfaction', and for the purposes of this paper these words should be regarded as synonyms.

### Assessors

The pairs of forms were assessed by four different groups of assessors: 35 experienced medical school selectors (recruited with the assistance of the CHMS Admissions to Medical Schools Group), a convenience sample of 19 doctors known to SI, 22 medical students from UCL, and 20 psychology students from UCL (for details see Additional File 1). The student groups were included because of studies suggesting that students can sometimes be better assessors than experienced judges [8]. After judging the pairs of forms, assessors provided brief data on age, sex, experience of medicine and student selection, and how long the task had taken, and they completed brief assessments of their own personality (Big Five, empathy, and communicative ability).

*Note*: There is much potential for confusion in understanding the results of this study. In particular we would like to emphasise that when we refer to *assessors *we are referring to the individuals making judgements of the forms in 2005, and when we refer to doctors (and to pairs of doctors) we are referring to the individuals who applied to medical school in 1990, and whose UCCA forms were used in this study, and to the questionnaire responses made by the same individuals as practising doctors in 2002/3. In particular it should be noted that both the doctors and the assessors each completed a Big Five personality assessment, and each is analysed below.

## Results

The pairs of application forms were judged by 96 assessors, 48 of whom looked at Book 1 and 48 looked at Book 2. A chance null hypothesis predicts that random guessing would result in an assessor making an average of 10 correct judgements out of 20 (50%). In fact overall, of the 1920 judgements made by the 96 judges, 963 (50.2%) were correct, which is not significantly different from 50% (χ^2 ^= .018, 1df, P = .89).

**Figure 2:**
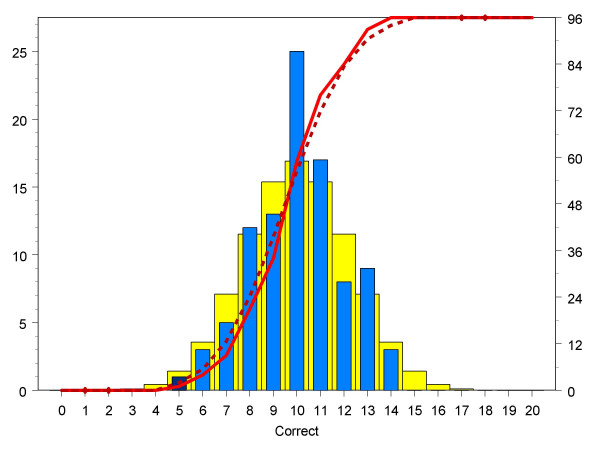
Number of happy doctors correctly identified by the 96 assessors, each of whom made a comparative judgement on 20 different pairs of doctors. The horizontal axis shows the number of times the doctor in a pair who was actually the happier was correctly identified as such by an assessor for each of the 20 pairs assessed by an assessor. The blue bars shows the actual numbers of correct judgements out of twenty for each assessor, and the yellow bars the expected distribution under a binomial distribution (left-hand vertical axis). The dashed red line shows the expected cumulative binomial distribution, and the solid line the actual cumulative binomial distribution (right-hand vertical axis). The light blue bars show pairs within the 95% range of the binomial distribution, whereas the dark blue bar show a pair which is outside the approximate 95% range, and hence is significant at about the 5% level.

The non-significant overall success rate may conceal differences between groups or individual assessors. The four groups of assessors showed similar overall success rates (medical school selectors: 48.0%; doctors 51.8%; medical students 51.4%; psychology students: 51.0% ; oneway ANOVA: F(3,92) = .932, P = .429). The chance expectations of individual assessors can be modelled using a binomial distribution (equivalent to asking how many heads would be expected if a fair coin was tossed 20 times). Figure 2 shows that the scores of individual assessors conform closely to binomial expectations. There is therefore no evidence that some individual assessors can carry out the task at significantly better than chance expectations. Neither was there evidence that the demographic or personality measures of the assessors predicted success at the task (see Additional File 1).

**Figure 3:**
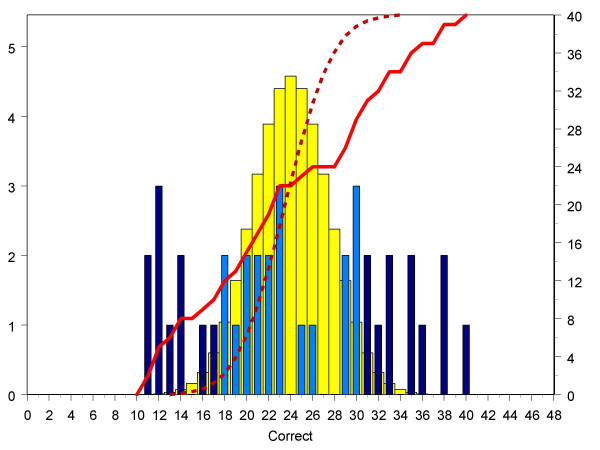
The number of times, for each of the 40 pairs of doctors, that the doctor in each pair of doctors who was actually more satisfied, was identified as happier by each of the 48 assessors who considered that pair of doctors. The horizontal axis shows the number of assessors (0–48) who were correct for a particular pair of doctors, and the light and dark blue bars shows the distribution for each pair of doctors. Note that in this figure the bars correspond to *pairs of doctors *(and hence sum to 40), whereas in figure 2 they corresponded to *assessors *(and hence sum to 96). The yellow bars show the expected distribution under a binomial distribution (left-hand vertical axis). The dashed red line shows the expected cumulative binomial distribution, and the solid line the actual cumulative binomial distribution (right-hand vertical axis). The light blue bars show pairs of doctors within the 95% range of the binomial distribution, whereas the dark blue bars show pairs which are outside the 95% range, and hence are significant at the 5% level.

A possible explanation for the inability of assessors to perform above chance expectations is that the task is simply too hard, and assessors are, in effect, forced to make decisions at random. That possibility can be evaluated by looking at the performance of the assessors on the 40 individual pairs of forms. Each pair of doctors was judged by 48 assessors, and therefore the binomial chance expectation is that on average the correct judgement for a particular pair of doctors will be made by 24 out of the 48 assessors. Figure 3 shows the actual number of correct judgements made by the assessors for each of the 40 pairs of doctors, and the binomial expectations (in effect the number of heads expected when a fair coin is tossed 48 times). The distribution is clearly non-binomial, with 21 out of the 40 pairs (52.5%) being outside of the 95% confidence interval expected by chance alone. However, although judgements for 21 of the pairs of doctors were non-random, in 11 pairs of doctors the assessors correctly predicted the happier doctor, but in the other 10 pairs the doctor chosen by the assessors as happier was actually the unhappier doctor.

Figure 3 confirms that assessors cannot be making random judgements, and that for many of the pairs of doctors there is a clear agreement between the assessors as to which doctor will be the happier (even though that judgement is erroneous for half of the pairs of doctors). A 'consensus score' was calculated to assess how often an individual assessor had concurred with the consensus judgement on the 21 pairs where on aggregate across all assessors there was agreement beyond chance expectations. Eighty-four of the assessors also reported how long they had taken to carry out the task (mean 80.8 minutes; SD 42.6 minutes; median = 60 minutes; range 30–240 minutes). Assessors who had taken longer to carry out the task were *less *likely to be correct overall (Pearson's r = -.289, p = .008) and *more *likely to concur with other judges (r = .274, p = .012), both correlations with time remaining significant after partialling out the other measure.

It is clear that assessors are unable to carry out the task beyond chance expectations, but they nevertheless concur with one another in their judgements. The question therefore arises as to the nature of the information from the forms which is acting as the basis for the assessors' (erroneous) consensus. We had background measures on the doctors in the pairs and we therefore looked to see whether those measures predicted the judgements of the assessors. It should be emphasised here that we are not looking here at correlations between the *actual happiness *of doctors and the background measures, but rather between those same background measures and which doctors are *thought more likely to be the happier by the assessors*. Although personality is known to be a strong correlate of happiness and satisfaction in doctors [3,7], there was no correlation between the difference in Big Five personality scores of the two doctors in each pair and the consensus judgement of the assessors (be it right or wrong). Assessors are not therefore implicitly assessing aspects of personality. We also had measures of educational achievement in the pairs of doctors, including O-level/GCSE grades, A-level grades, and expected A-level grades in those applying pre-A-level. The difference in educational achievement between the doctors in a pair correlated significantly with the consensus judgement of the assessors, with the largest correlation being with expected A-level grades (r = .321, p = .011, N = 62). Since expected A-level grades, and other aspects of educational achievement, are often referred to in referees' reports, it seems likely that assessors are judging that the doctor with the higher, predicted educational achievement will be the happier, more satisfied doctor.

## Discussion

UK medical schools put extensive effort into reading the application forms submitted by applicants, using them as a basis for shortlisting for interview (and in some cases for making offers), and assessing both academic achievement and motivation from them. However the present study suggests that assessors, including experienced medical school selectors, are unable to use application forms to judge which applicants will become happy and satisfied doctors. That does not mean, though, that selectors are making random judgements on the basis of the forms. There is a consensus between assessors, which is greatest amongst those who have spent longest reading the forms. However that consensus seems principally to result from using information about the academic potential of applicants, despite there being good reason to believe that it is not a lack of academic ability which causes the dissatisfaction of unhappy doctors, or that academic ability correlates with happiness [3]. In view of the implications of the present findings for current methods of student selection in the UK (and elsewhere), it would be reassuring if other studies, in other settings, and with different outcome criteria, were to find similar results, and we hope that such studies will be carried out.

The implicit use of academic ability as a criterion is consistent with other work [4], which has shown that when raters used a structured pro-forma to assess the presence of various attributes in a resume (e.g., academic ability, social exchanges), then those ratings are related to applicants personality and cognitive abilities [9]. However, these results consider judgements based on pre-defined categories, whereas personal statements are often judged without such a standardized approach – as in UCCA and UCAS forms – and therefore the predictive validity of personal statements for identifying 'at risk' candidates is low. Even should structured personal statement coding be correlated with personality, it might still be more effective to use psychometrically assessed personality scores than personal statements, if it were desired to select on the basis of personality. Such conclusions are, of course, predicated on the basis that selectors use personal statements to identify qualities that mark whether a candidate will be a happy and good doctor. The analyses reported here suggest, though, that academic criteria are still relied on as the main differentiator, which may reflect social and cultural stereotypes of what makes a good doctor.

The overall outcome measure in our study, of satisfaction and happiness with medicine as a career, might be criticised on the grounds that what is really required is knowledgeable, effective, doctors who stay in post. If knowledgeable, academically well-qualified doctors alone is all that is required, then probably the best predictor of academic success is educational achievement [3,10], but that is assessed most efficiently, effectively and objectively from measures of educational achievement, rather than indirectly from a referee's report. Academic ability and career satisfaction are not however correlated [3]. There is however good evidence that our unhappy doctors are stressed and burned out (see Additional File 1), that stress and burnout result in less effective work in doctors [11], and that dissatisfied, unhappy doctors are the ones who are most likely to want to leave medicine [1]. Satisfaction is therefore an important outcome variable.

Perhaps the most surprising thing about our study is that the task was completely impossible. That might reflect the relative poverty of the information provided on the application forms (for the personal statement the median length is 107 words (mean = 106, SD 22, quartiles 92–120, range = 54–162), and for referees' reports the median length is 398 words (mean = 391, SD 111; quartiles 325–465; range 114–734)). The information provided might also be inaccurate or misleading, either unintentionally, or as part of a process of 'impression management' [5]. Referees have an obligation to the medical school, but also to the person on whose behalf they are making a statement, perhaps ignoring their weaknesses and over-emphasising their strengths, so that some geese appear as swans. It is therefore possible that information obtained differently might be more effective in prediction. The UCCA forms completed in 1990 are now somewhat different from those currently used, and it is possible that modern forms might provide more information which is more useful. However several selectors spontaneously commented that they were surprised by the honesty and the openness of the referees' reports made in 1990, and that more modern statements seemed bland, non-committal and uninformative in comparison. Our results are therefore likely to be valid for modern application forms.

We had wondered whether there may be further information about applicants concealed within the subtleties and the nuances of the language used (e.g. the use of negative versus positive emotion words). We therefore used a computerised text analysis program, LIWC [12,13], to assess personal statements and referees' reports on about 70 semantic, syntactic and stylistic measures, but could find no systematic difference between UCCA forms from happy and unhappy doctors. Nevertheless it is possible that there is additional information within the specific content of the forms, which could be indirect indicators of personality, and in particular neuroticism, which is itself a major predictor of stress and burnout.

The Schwartz Report into admissions to universities, was critical of the use of selection methods for which there was not demonstrable evidence of reliability or validity [14]. UCCA/UCAS forms, along with A-level grades, are the single most important piece of evidence used in medical school selection, each being carefully read and assessed, and often they are the sole basis for rejection. Applicants, their parents, and their schools therefore put much effort into every detail of the forms. Our study is an assessment of the long-term validity of judgements made from the information on UCCA/UCAS forms. Judgements made are to some extent reliable, as seen by the consensus between assessors (and shown elsewhere [15]). However although many claims are made for the utility of the personal and referee's information provided on the forms, we could find no evidence of long-term predictive validity for an important outcome variable – the judgement of whether or not an applicant will be a happy and satisfied doctor, or instead will be an unhappy, stressed, burned out, dissatisfied doctor who does not enjoy their job and thinks often of leaving for another career.

## Competing interests

The author(s) declare that they have no competing interests.

## Authors' contributions

The 1991 cohort study was originated by ICM. ICM and EF had the idea of transcribing and analysing UCCA forms, and the forms were scanned and transcribed by JL and EF. ICM designed the experimental study, and SI and AC carried out the experiment as a part of research projects submitted as a component of their degree programmes. ICM wrote the first draft of the manuscript, and all authors revised the manuscript. ICM is the guarantor for the study.

## Pre-publication history

The pre-publication history for this paper can be accessed here:



## Supplementary Material

Additional File 1 Supplementary Information – three additional tables and one additional figure Click here for file
