# Supplementary material for: Unhappiness and dissatisfaction in doctors cannot be predicted by selectors from medical school application forms: A prospective, longitudinal study
Source: BMC Med Educ. 2005 Dec 13;5:38. doi: 10.1186/1472-6920-5-38 (PMC1325230; doi:10.1186/1472-6920-5-38)

Supplementary Information

Contents:

Table S1: Comparison of Happy/Satisfied doctors with Unhappy/Dissatisfied doctors on measures of stress, burnout and the Big Five personality measures, and on educational achievement.

Table S2: Age and sex distributions of the assessors.

Table S3: Correlation of personality and background measures *of assessors* with number of correct judgements and with mean strength of judgements.

Figure S1: The correlation of the consensus measure with the time taken to carry out the study.

Table S1: Comparison of Happy/Satisfied doctors with Unhappy/Dissatisfied doctors on measures of stress, burnout and the Big Five personality measures. N=40 for most measures, except for occasional missing values.

	Unhappy/Dissatisfied		Happy/Satisfied		
	Mean	SD	Mean	SD	Significance
Composite Satisfaction measure	5.27	2.58	16.95	.59	n/a
Abbreviated Maslach Burnout Inventory					
Emotional exhaustion	12.35	3.32	5.77	3.33	P<.001
Depersonalisation	8.69	4.61	4.40	4.06	P<.001
Personal accomplishment	11.31	3.34	15.52	2.66	P<.001
Stress levels (General Health Questionnaire)					
GHQ-12 (0-1-2-3 scoring)	14.82	5.00	9.15	3.86	P<.001
GHQ-12 caseness (≥ 4 on 0-0-1-1scoring)	19/40=47.5%		3/40=7.5%		P<.001
Abbreviated Big Five Personality scale					
Neuroticism	9.75	2.13	7.3	2.06	P<.001
Extraversion	8.70	2.02	10.57	1.86	P<.001
Openness to experience	9.77	2.99	10.95	2.47	P=.059
Agreeableness	12.40	1.39	13.25	1.53	P=.011
Conscientiousness	10.67	1.74	11.45	1.74	P=.050
Educational achievement					
Mean A-level grade (A=5, E=1)	4.35	.50	4.35	.56	P=.989
Mean estimated A-level grade	4.52	.53	4.61	.40	P=.458
Mean GCSE grade	4.62	.36	4.64	.35	P=.868
Number of GCSEs	9.20	1.04	8.35	2.94	P=.090

NB A significance test is not meaningful for the composite score since the groups were formed on the basis of extreme scores on this measure.

Table S2: Age and sex distribution of the assessors.

Group	Age group					Sex		Total
	20-29	30-39	40-49	50-59	60+	Male	Female	
Medical school selectors	1	4	10	17	3	19	16	35
Doctors	7	4	2	4	2	9	10	19
Medical students	22	0	0	0	0	9	13	22
Psychology students	20	0	0	0	0	7	13	20
Total	50	8	12	21	5	44	52	96

Table S3: Correlation of personality and background measures *of assessors* with number of correct judgements and with mean strength of judgements (proportion of 'Definite' answers). Note that only three of the 28 correlations are significant at the 5% level (shown in bold), and none are significant at the 1% level. A Bonferroni correction would mean that none of the correlations is significant. N=96 for sex and age, and 84 for most personality correlations, except for occasional missing values.

	Correlation with number of correct judgements	Correlation with number of 'definite' judgements
Demographic factors		
Sex	r = .015 NS	r =-.032 NS
Age	r = -.215 **P=.035**	r = .058 NS
Abbreviated Big Five Personality scale		
Neuroticism	r = -.014 NS	r = -.067 NS
Extraversion	r = .097 NS	r = -.013 NS
Openness to experience	r =.180 NS	**r = -.216 P=.040**
Agreeableness	r = .042 NS	r = -.110 NS
Conscientiousness	r = -.165 NS	r =.049 NS
Abbreviated empathy scale		
Fantasy	r = .131 NS	r = -.182 NS
Perspective-taking	r = .146 NS	r = .069 NS
Empathic concern	r = .050 NS	**r = .231 P=.028**
Personal distress	r = .038 NS	r = -.134 NS
Communicative Skills		
Non-verbal communication	r = .121 NS	r = -.108 NS
Effective communication	r = .032 NS	r = .111 NS
Dominant communication	r = -.041 NS	r = .180 NS

Figure S1. The correlation of the consensus measure with the time taken to carry out the study. The consensus measure was calculated only for those pairs of doctors for which there was a significant consensus (see figure 3 in the main paper). Each assessor was given a score of +1 for a judgement which agreed with the consensus and -1 for any judgement which disagreed with the consensus. Because there were slightly different numbers of consensus pairs in Book 1 and Book 2, the scores were then standardised so that a score of +100 was given for complete agreement with the consensus, and a score of zero was given for no average agreement with consensus (i.e. there were equal numbers of scores of +1 and -1). The graph shows the relationship between the consensus score and the reported time to complete the questionnaire. The red line shows a lowess curve, to indicate the underlying trend.

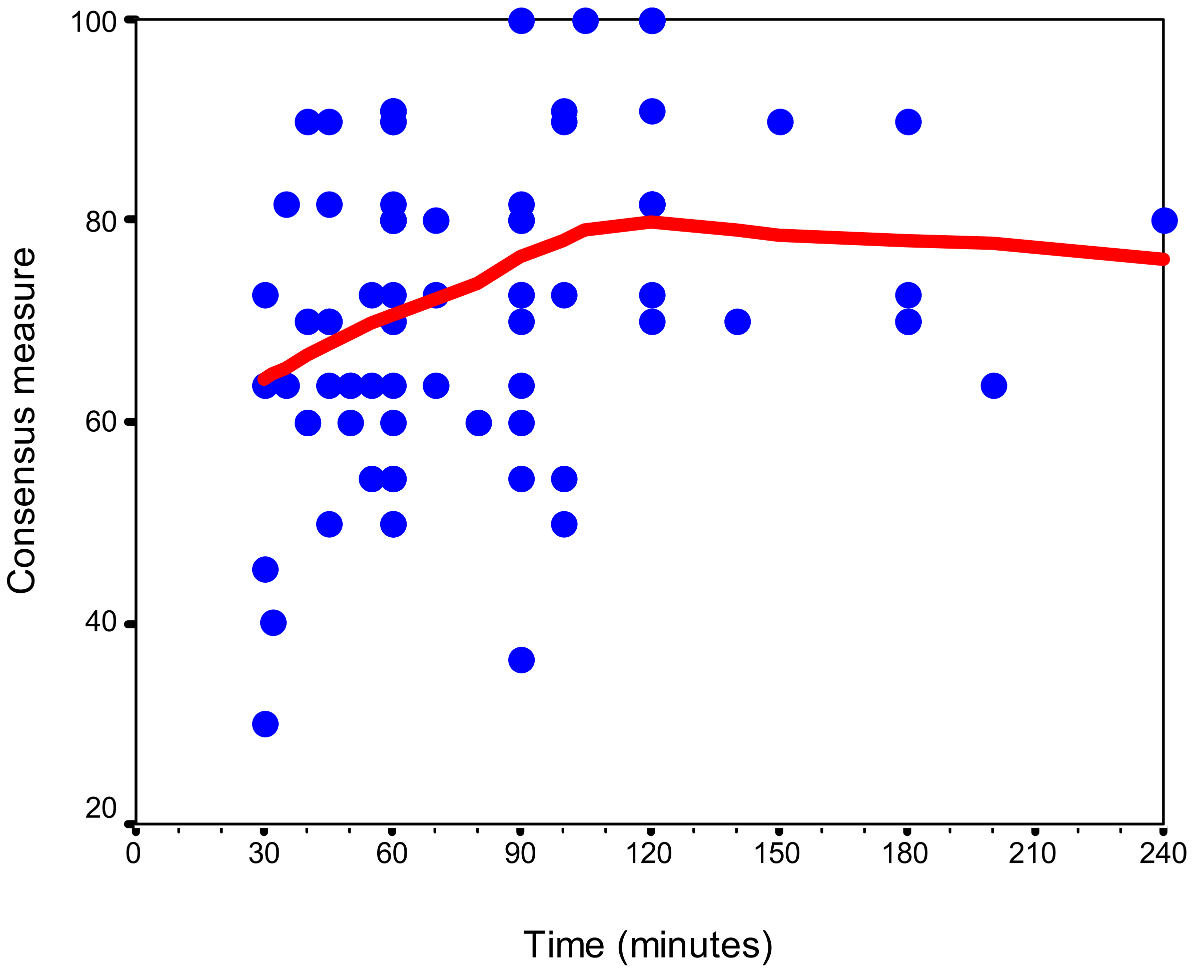

Supplement: Additional File 1 — Supplementary Information – three additional tables and one additional figure [file 1472-6920-5-38-S1.pdf]
